# Multi-ciliated microswimmers–metachronal coordination and helical swimming

**DOI:** 10.1140/epje/s10189-021-00078-x

**Published:** 2021-06-08

**Authors:** Sebastian Rode, Jens Elgeti, Gerhard Gompper

**Affiliations:** Theoretical Physics of Living Matter, Institute of Biological Information Processing and Institute for Advanced Simulation, Forschungszentrum Jülich, 52425 Jülich, Germany

## Abstract

**Abstract:**

The dynamics and motion of multi-ciliated microswimmers with a spherical body and a small number *N* (with $5< N < 60$) of cilia with length comparable to the body radius, is investigated by mesoscale hydrodynamics simulations. A metachronal wave is imposed for the cilia beat, for which the wave vector has both a longitudinal and a latitudinal component. The dynamics and motion is characterized by the swimming velocity, its variation over the beat cycle, the spinning velocity around the main body axis, as well as the parameters of the helical trajectory. Our simulation results show that the microswimmer motion strongly depends on the latitudinal wave number and the longitudinal phase lag. The microswimmers are found to swim smoothly and usually spin around their own axis. Chirality of the metachronal beat pattern generically generates helical trajectories. In most cases, the helices are thin and stretched, i.e., the helix radius is about an order of magnitude smaller than the pitch. The rotational diffusion of the microswimmer is significantly smaller than the passive rotational diffusion of the body alone, which indicates that the extended cilia contribute strongly to the hydrodynamic radius. The swimming velocity is found to increase with the cilia number *N* with a slightly sublinear power law, consistent with the behavior expected from the dependence of the transport velocity of planar cilia arrays on the cilia separation.

**Graphic abstract:**

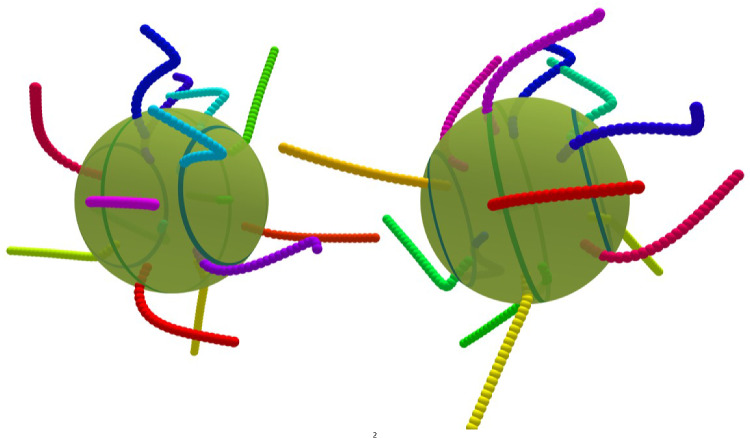

**Supplementary Information:**

The online version contains supplementary material available at 10.1140/epje/s10189-021-00078-x.

## Introduction

Cilia and flagella are the ubiquitous machinery in eukaryotic cells and organisms to generate fluid flow and to propel cells and microorganisms in a fluid environment [1, 2]. While many eukaryotic flagella have the beat pattern of a sinusoidal traveling wave and are usually employed to propel single cells like sperm [3], cilia typically have two distinct phases in their beat cycle—the power and the recovery stroke—and often work together in pairs like in Chlamydomonas reinhardtii [4], or in large cilia carpets. Examples for the concerted action of cilia in carpets are the transport of mucus in the airways [5, 6], the flow generation of the cerebrospinal fluid in brain ventricles [7–9], and the swimming of multi-ciliated microorganisms, such as the green alga *Volvox* [10], the protozoan *Paramecium* [11], and the placidozoan *Opalina* [12].

A remarkable feature of cilia carpets is that the beat is highly coordinated in the form of metachronal waves [12–17], where the beat cycles of neighboring cilia have a fixed phase shift The power-stroke direction can be parallel or antiparallel to the propagation direction of the metachronal wave, which is denoted as symplectic or antiplectic wave, but can also point right-wise or left-wise from the wave direction, which is denoted dexioplectic or laeoplectic wave. The latter wave form clearly requires some chirality in the system, which can either be in the aplanarity of the ciliary beat [18], or result from the spatial arrangement of cilia.

The origin and effect on the transport efficiency of the ciliary beat and of the metachronal wave is fascinating and has thus been investigated intensively. It is now well established that hydrodynamic interactions are strong enough to cause coordination between neighboring cilia [19–23] and suffice to explain the formation of metachronal waves in cilia arrays [17]. It has also been shown that the transport efficiency can be much higher than for perfect beat synchronization, related to the fact that always a fraction of the cilia is in the power stroke, thus avoiding a forward-backward motion of the fluid. Additionally, flow generated by the power stroke at the wave crest experiences only small resistance from cilia in the neighboring wave trough, which can all remain close to the anchoring surface during the recovery stroke—both without much steric hindrance [14, 17, 18]. A further interesting issue is the coordination of the beat directions in cilia carpets, which is now believed to be a self-organized process mediated by the fluid flow [6, 7, 14, 22, 23]. On the other hand, the synchronization of the beat of the two flagella of Chlamydomonas reinhardtii arise from an elastic mechanical coupling at their basal foot [24, 25].

More complex is the cilia coordination in multi-ciliated spherical or spheroidal microorganisms. One reason is the well-know “hairy-ball” theorem, which states that there is no non-vanishing continuous tangent vector field on a surface of spherical topology [26]. This implies that the power-stroke directions of neighboring cilia cannot be parallel everywhere on a spherical surface, but there have to be at least two defects, which can either be of hedgehog or of swirl type. A second reason is that plane metachronal waves are not possible on curved surfaces.

Volvox is a perfect model system for experimental studies of swimming and cilia synchronization [27–29]. These studies reveal the existence of a symplectic metachronal wave [27], and that the average metachronal coordination is punctuated by periodic phase defects during which synchrony is partial and limited to specific groups of cells [28]. Under conditions of decreasing nutrient concentration, Volvox colonies were found to grow larger and increase their flagellar length, separating the somatic cells further, with the opposing effects of increasing beating force and flagellar spacing balance, not significantly affecting the fluid speed at the colony surface [29].

The theoretical description of the swimming of multi-ciliated microorganisms has lead to the early development of the squirmer model [30, 31], in which the effect of the ciliary beating is mimicked by a prescribed surface velocity. This model has been generalized more recently to spheriodal shapes [32] and is employed nowadays to describe the collective swimming behavior of many types of microswimmers [1, 33]. The squirmer model has also been generalized to capture the effect of metachronal waves and to include azimuthal swirl on the continuum level [34]. This model predicts mean swimming speeds and angular velocities as a function of the colony radius qualitatively correct, but underestimates both velocities quantitatively [34].

The squirmer model applies in the limit that the cilia length and the separation of their anchoring points on the surface is much smaller than the body size. The dynamics and flow generation of individual cilia becomes more important in the opposite limit. A model with explicit cilia and a prescribed metachronal wave has been introduced recently [35, 36]. The model facilitates the calculation of hydrodynamic interactions between cilia and the cell body under free-swimming conditions. An antiplectic metachronal wave is predicted to be optimal in the swimming speed with various cell-body aspect ratios, which is consistent with former theoretical studies [15, 37]. The swimming velocity of model ciliates is found to be well represented by the squirmer model. The effect of oblique wave propagation is also briefly touched and is found to lead to a helical swimming trajectory [35]. Further analysis of this model showed that for constant number of cilia, the swimming velocity scales with the inverse body radius squared, which is explained by solving the axisymmetric Stokes equation around the squirmer [36]; this implies a cilia density for optimal swimming velocity, which nearly independent of the swimmer radius.

**Fig. 1:**
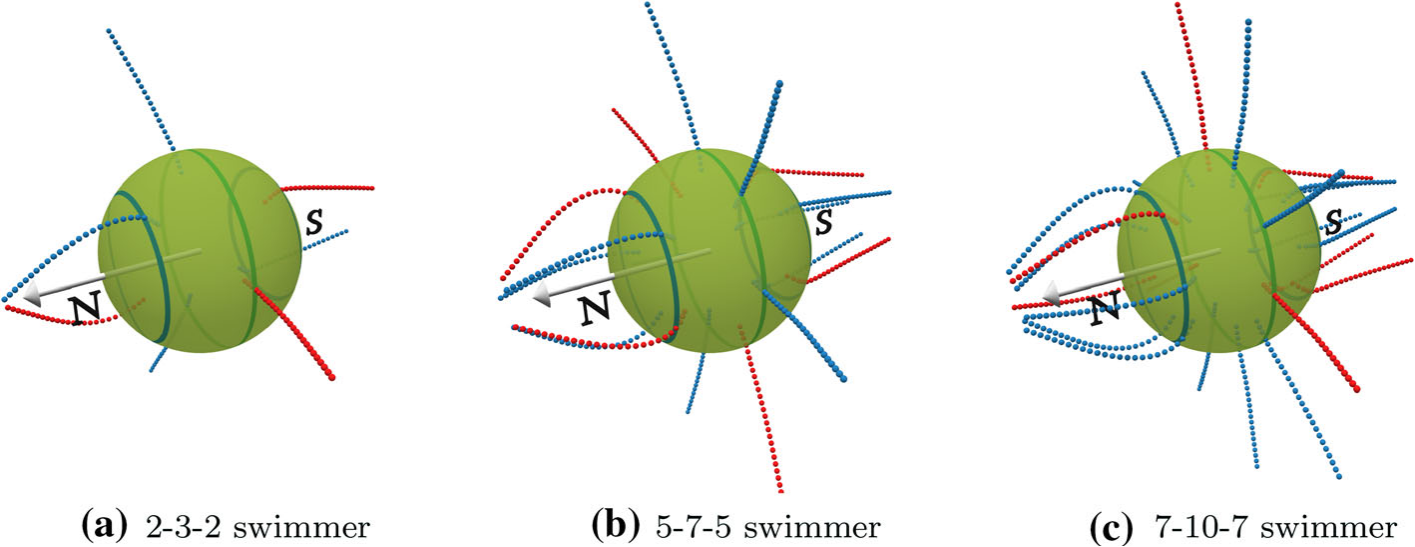
Three swimmers with an antiplectic metachronal wave of phase lag $\chi =-77^\circ$ with different numbers of cilia placed at the equator, with $\theta =0.0$, and two rings at $\theta =\pm 45^\circ$

Also, there has been significant progress recently on the experimental techniques for cilia characterization [38], as well as the construction of artificial, externally actuated cilia carpets [39], which relates to the goal of the construction of soft microbots [40]. Soft robots with antiplectic waves have been shown to exhibit much higher locomotion speed than those with symplectic waves [39].

We employ a similar model of ciliated microorganisms as studied in Ref. [35], but focus on the regime of a smaller number of longer cilia—comparable in length to the body radius. Volvocalean algae which falls into this parameter range are, for example, the 16-celled *Pandorina* and the 32-celled Eudorina [29, 41]. We focus on the efficiency of metachronal waves for translational and rotational motion of the swimmer. In particular, we investigate the influence of the wave direction, the phase lag between neighboring cilia, and the cilia density, on the swimming efficiency and the persistence of the swimming trajectory. In particular, we identify parameter combinations for which helical swimming trajectories emerge.

## Ciliated microswimmer and hydrodynamic simulation

The ciliated microswimmer is modeled as a spherical body of radius *R*, to which several cilia are attached (see Fig. 1). The body consists of $N_b$ point particles, which are covering the surface of a sphere homogeneously, with an additional particle at its center. The particles on the surface are connected to their neighbors by stiff harmonic springs to form a triangular network, as well as to the center particle to form an essentially rigid sphere. Two points on the surface, which lie on a straight line through the center, are selected to define a body-fixed polar axis.

Following Refs. [17, 42], we model a cilium of length *L* by three semi-flexible polymers, which are arranged on the surface of a (hypothetical) cylinder with triangular cross section. Each semi-flexible polymer is described by a bead-spring model, consisting of $N_c$ beads connected by harmonic springs with rest length $\ell _c$ and spring constant $k_c$. The three polymers are interconnected by springs in order to retain the cylindrical shape over time. The cilia are anchored in the body with a “clamped” boundary condition, which is achieved by extending the filament by a segment of length (3/16)*R* inside the body. This anchoring is implemented by stiff springs which connect the sub-surface part of the cilium to the sphere surface, mimicking the embedding of the basal bodies of the cilia in the cell body. In detail, the first and fourth particle of each of the three polymers constituting the cilium are connected to the closest particle on the sphere surface as well as its next-nearest neighbors. This construction anchors the cilium tightly to the body without a noticeable body deformation—even for the largest applied ciliary forces.

**Fig. 2:**
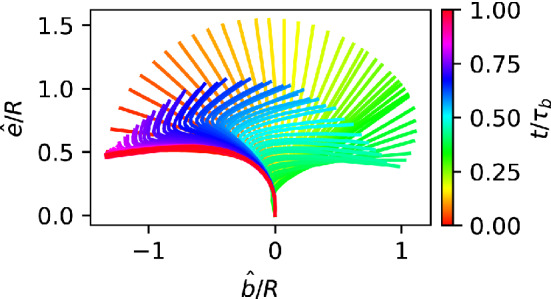
Sketch of the ciliary beat pattern—The color indicates time. The elongated conformation during the power stroke (orange to green) is followed by the buckled conformation during the recovery stroke (green to blue)

The cilia beat pattern consists of a power and a recovery stroke (see Fig. 2). The ciliary beat is generated by varying the equilibrium spring lengths $\ell _c$ of one selected polymer, both spatially along the cilium and periodically in time, which creates a spatially and temporarily varying cilium curvature. The selection of the active polymer defines the beat plane. The beat pattern of power and recovery stoke is obtained by prescribing an analytic function for the desired local curvature of the cilium. For details, see Appendix A.. This is inspired by the molecular mechanism which drives ciliary beating, where molecular motors apply torques along the flagellum, but each motor has a maximum force it can generate. To mimic the stall force, and to avoid artificial cilia shapes due to unnaturally large local torques, we limit the change of equilibrium bond length such that a maximum energy of $1.0~k_B T$ per MPC time step (see below) can be inserted into the system, where $k_BT$ is the thermal energy.

The dynamics of the beat is not only determined by the time-dependence of the internal torques, but is also affected by the flow field around the swimmer and the elastic properties of the cilium. To model the hydrodynamics of the embedding fluid, we employ multi-particle collision dynamics (MPC) [43, 44], a mesoscale simulation technique, which is ideally suited for simulations with a particle-based model of an active microswimmer. In this approach, the fluid consists of point particles, each characterized by its location $\mathbf{r}_i(t)$ and velocity $\mathbf{v}_i(t)$. These particles move ballistically during the streaming step for a time interval *h*. In the subsequent collision step, all particles are sorted into the cells of a simple cubic lattice with lattice constant *a*. Particles in each cell interact by exchanging momentum, but in such a way to conserve the mass and linear momentum within each cell. A cell-level canonical thermostat (with Maxwell-Boltzmann scaling) is applied after every collision step to maintain a constant temperature *T* [45].

The mechano-elastic model of the ciliated microswimmer is coupled to the fluid in the collision step. This is done by sorting all point particles, which constitute the swimmer, into the same collision cell as the fluid particles, and including them into the momentum exchange. Here, it has been shown that a good hydrodynamic coupling is obtained then the mass *M* of the swimmer particles is significantly larger than the mass *m* of the fluid particles, such that $M=\rho a^3 m$, where $\rho$ is the average number of fluid particles [46]. This type of hydrodynamic modeling has been tested and verified carefully, for example by studying the hydrodynamic diffusion of spheres with the meshed surface described above, which showed very good agreement with the Stokes–Einstein results for spheres with no-slip boundary conditions [47], or the swimming velocity of a sinusoidally beating flagellum in 2D, which showed excellent agreement with analytical results for the Taylor sheet [48]. A detailed description of the MPC technique and a review of its application to many systems in soft, active and living matter is provided in Refs. [43, 44, 49, 50].

For the investigation of the self-organization of beating cilia arrays into metachronal waves in Ref. [17], the switch from power to recovery stroke, and back, needs triggers, which are reached faster or slower depending on the environmental conditions (“geometrical clutch” hypothesis [51]). In Ref. [17], a critical value curvature of the cilium, and a maximum angle between the extended cilium and the normal vector to the cell surface, were employed to trigger these switching events. For the major part of our study, we employ a variant of this model, in which the force generation is modified such that the switch between power and recovery stroke becomes deterministic in time, with a power-stroke time $\tau _p$ and a recovery-stroke time $\tau _r$, with $\tau _r > \tau _b$, which results in a constant beat period $\tau _b = \tau _p + \tau _r$. In this case, a phase lag between the beats of neighboring cilia is imposed to induce a metachronal wave (see below).

We consider a ciliated microswimmer, where a total of *N* cilia are placed equidistantly on three latitudinal rings. In spherical coordinates $(\varphi , \theta )$, one ring is located at the equator, $\theta =0$, and two rings at $\theta =\pm 45^\circ$, see Fig. 1. In order to have a roughly constant cilia density, the number of cilia at the “polar circles” is reduced by a factor close to $1/\cos \theta = \sqrt{2}$ compared to their number $N_{\mathrm{eq}}$ at the equator. The “first” cilium on each ring is initially placed at $\varphi =0$; subsequently, the cilia positions on both polar rings are shifted azimuthally in the same direction by a small $\Delta \varphi = 180^\circ /N_{\mathrm{eq}}$ to avoid perfect registry for one particular longitude. Several examples of microswimmer with various numbers of cilia are shown in Fig. 1).

The power-stroke direction of the cilia can be along $\mathbf{e}_\theta$, i.e., be parallel to the circles of longitude, or deviate from this highly symmetric case. This is modeled by rotating the beat plane of each cilium around the radial axis $\mathbf{e}_r$ by an angle $\theta _r$ (with $\theta _r=0$ corresponding to the longitudinal beat direction). A beat-plane orientation with $\theta _r \ne 0$ introduces chirality in the swimmer propulsion pattern.

In the case of an imposed metachronal wave, we define a local phase $\Psi (\varphi , \theta )$ for each cilium on the surface of the sphere,

1 $$\begin{aligned} \Psi (\varphi , \theta ) = k_\varphi \varphi + k_\theta \theta , \end{aligned}$$

with $-90^\circ< \theta < +90^\circ$ and $0\le \varphi < 360^\circ$, where the direction of the wave is determined by the wave vector $\mathbf{k}=(k_\varphi , k_\theta )$, which has longitudinal and latitudinal components, $k_\theta$ and $k_\varphi$, respectively. Since we want to have continuous wave solution traveling around the circles of constant latitude, $k_\varphi$ has to be an integer number. For $N_{\mathrm{eq}}$ equally-spaced cilia on the equator, $k_\varphi =N_{\mathrm{eq}}/2$ results in a phase lag between neighboring cilia of $\Delta \Psi = 180^\circ$. Since $k_\varphi \in {\mathbb {N}}$, we limit it to $k_\varphi =0,1,...,(N_{\mathrm{eq}}//2+1)$, where // denotes integer division. The longitudinal wave vector $k_\theta \in {\mathbb {R}}$ is not restricted by any physical boundary conditions. We employ the phase lag

2 $$\begin{aligned} \chi = k_\theta \, \Delta \theta = k_\theta \cdot \, 45^\circ , \end{aligned}$$

between neighboring rings to quantify $k_\theta$, where $\Delta \theta =45^\circ$ is the fixed longitudinal angular distance between successive rings.

For zero latitudinal wave component, $k_\varphi =0$, a positive phase lag $\chi$ corresponds to a symplectic metachronal wave, which travels in the direction of the power stroke, a negative phase lag $\chi$ to an antiplectic metachronal wave, which travels in the direction of the recovery stroke. For non-zero latitudinal component $k_\varphi$, the metachronal wave becomes either dexioplectic ($k_\theta > 0$) or laeoplectic ($k_\theta < 0$). Because the microswimmer is constructed essentially symmetric to the main axis, dexioplectic waves show the same effect on the propulsion as laeoplectic, except for an opposite direction of axial rotation $\Omega _n$. Therefore, we restrict our analysis to symplectic, antiplectic ($k_\varphi =0$) and dexioplectic ($k_\varphi > 0$) metachronal waves with varying phase lags $\chi$.

For simulations, we employ the following parameters. The spherical body consists of $N_b=643$ mesh points, the cilia are constructed with $N_c=26$ beads for each polymer strand, they have length (outside the body) of $L=1.375\,R$, and the body radius in terms of MPC collision box size *a* is $R=8a$, which guarantees a good resolution of the hydrodynamic flow fields. The MPC simulations employ collisions described by stochastic rotation dynamics, with a time step $h=0.05$, cell size $a=1$, rotation angle $\alpha _0= 130^\circ$, number of fluid particles per cell $\rho a^3=10$, and an overall simulation box size of $(100a)^3$. With the typical parameters employed for the ciliary beat, we obtain the Reynolds number for the microswimmer motion, $Re_s= \rho \langle v \rangle R / \eta _f \simeq \rho R^2/(\tau \eta _f) = 0.2$, and the Reynolds number for the cilia beat, $Re_c = \rho L_c^2 /(\tau \eta _f) = 0.4$, where $\eta _f$ is the fluid viscosity. As both Reynolds numbers are significantly less than unity, we conclude that inertia effects play at most a minor role.

## Results

### The 5-7-5 swimmer with longitudinal beat direction

**Fig. 3:**
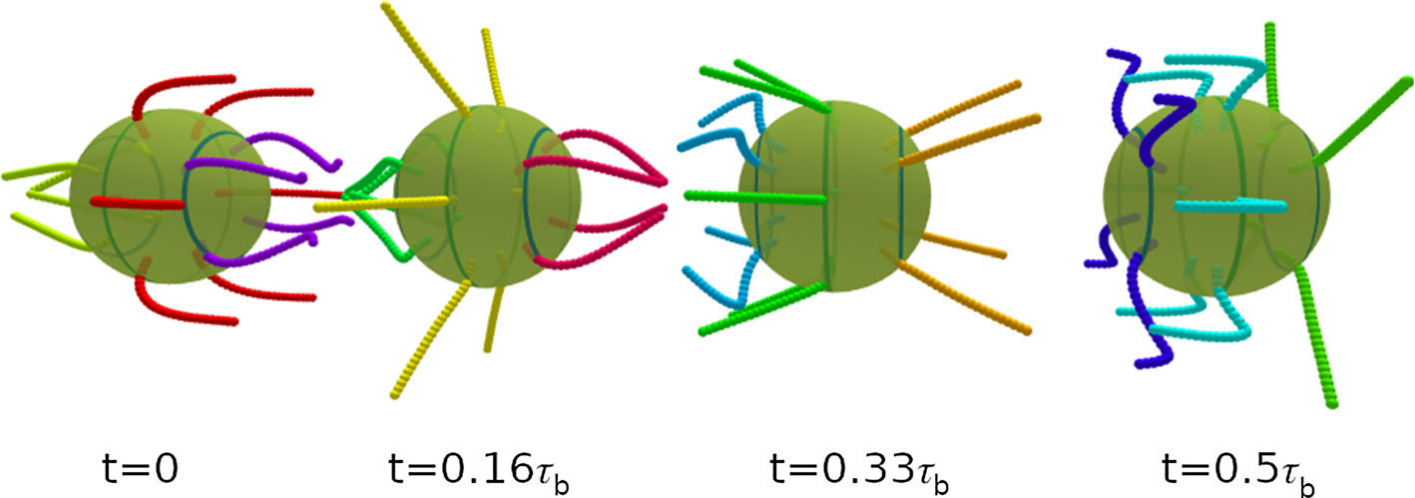
Temporal beat pattern of a 4-6-4 swimmer with cilia beat in the longitudinal direction ($\theta _r=0$), with $k_\varphi =0$ and phase lag $\chi =-77^\circ$. The varying cilium color indicates the instantaneous stage in the beat cycle (compare Fig. 2). The progression of the beat is indicated by the time *t*, given in units of the beat period $\tau _b$. The motion of the swimmer is from left to right. The translational motion is not to scale. See also movie SM1 for an illustration of the swimming behavior

**Fig. 4:**
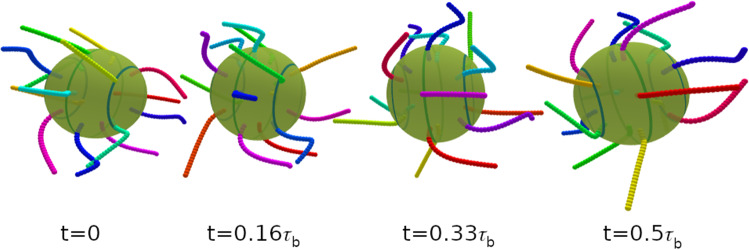
Temporal beat pattern of a 4-6-4 swimmer with cilia beat in the longitudinal direction ($\theta _r=0$), with $k_\varphi =1$ and phase lag $\chi =-77^\circ$. The varying cilium color indicates the instantaneous stage in the beat cycle (compare Fig. 2). The progression of the beat is indicated by the time *t*, given in units of the beat period $\tau _b$. The motion of the swimmer is from left to right. The translational motion is not to scale. See also movie SM2 for an illustration of the swimming behavior

We focus on the swimming properties of a spherical swimmer with 7 cilia on the equator and 5 cilia on the two polar rings see Fig. 1, with power stroke direction along the main body axis, $\theta _r=0$. Examples for the beating dynamics with phase lag $\chi =-77^\circ$ with latitudinal wave numbers $k_\varphi =0$ and $k_\varphi =1$ are shown in Figs. 3 and  4, respectively.

**Fig. 5:**
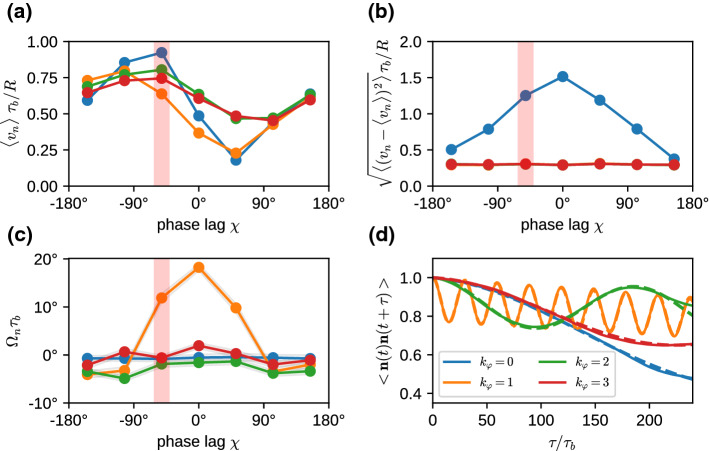
Swimming properties of a 5-7-5 swimmer with cilia beat in the longitudinal direction ($\theta _r=0$), for various phase lags $\chi$ and latitudinal modes $k_\varphi$. **a** Average swimming velocity $<v_n>$. **b** Velocity fluctuations $\sqrt{<(v_n - <v_n>)^2>}$ around the average. **c** Rotational velocity $\Omega _n$ around the main body axis. **d** Temporal auto-correlation function $<\mathbf{n}(t)\cdot \mathbf{n}(t+\tau )>_t$ for swimmers with phase lag $\chi =-50^\circ$ indicated by red stripe in (a-c). The dashed lines are fits to the auto-correlation function, Eq. (6) (see text). Note that in **(b)**, the curves for $k_\varphi =1$ and $k_\varphi =2$ are numerically identical to the curve for $k_\varphi =3$ and are therefore not visible

We consider the propulsion velocity $\langle v_n \rangle$, the velocity fluctuations $\sqrt{\langle (v_n - \langle v_n \rangle )^2 \rangle }$ around the average, and the rotational velocity $\Omega _n$ around the main body axis, see Fig. 5. All these quantities depend on both the phase lag $\chi$ in the longitudinal direction and the wave number $k_\varphi$ in the latitudinal direction. The propulsion velocity $\langle v_n \rangle$ in the direction of the main body axis shows a pronounced “sinusoidal” dependence on the phase lag $\chi$ for all wave numbers $k_\varphi$, see Fig. 5a. Here, the strongest variation is found for a metachronal wave with $k_\varphi =0$. The swimming velocity $v_n$ is maximal for a negative phase lag of $\chi \simeq -50^\circ$ (see Fig. 5a) and reaches almost a body length per beat cycle, $v_n = 0.9 R/\tau _b$. Such a negative phase lag corresponds to an antiplectic metachronal wave, with cilia of one ring lagging behind those of the subsequent ring by a little bit more than 1/4 of a beat cycle, where the wave travels against the direction of the power stroke. The smallest velocity of $v_n = 0.2 R/\tau _b$ corresponds to symplectic wave with a positive phase lag of $\chi \simeq 50^\circ$, where the wave travels with the direction of the power stroke. These results are consistent with former theoretical studies of efficiency optimization [15, 16].

The variation $\sqrt{\langle (v_n - \langle v_n \rangle )^2 \rangle }$ of the swimming velocity is shown in Fig. 5b. The swimming velocity $v_n$ fluctuates mostly for swimmers with $k_\varphi =0$, whereas it is nearly independent of the phase lag $\chi$ for latitudinal wave numbers with $k_\varphi >0$. In particular for the synchronous case, with $\chi =0$, the swimmer moves quickly forward during the power stroke, but reverses its direction of motion during the recovery stroke, which in sum leads to a relative slow average velocity with high fluctuations.

For $k_\varphi \ge 1$, the latitudinal component of the wave leads to an additional rotation velocity $\Omega _n$ around its main body axis (see Fig. 5c). The rotation is most pronounced for $k_\varphi =1$ and $-50^\circ< \chi < 50^\circ$ and is very small for $k_\varphi \ge 2$ and all phase lags $\chi$. This behavior can be understood by considering two contributions. (i) For $k_\varphi \ge 1$, rotation is enhanced when the metachronal wave travels in the latitudinal direction, i.e., $\chi \simeq 0$. (ii) Latitudinal wave numbers $k_\varphi \ge 2$ imply short metachronal wave lengths, which cannot propel fluid effectively, because the opposing beat of neighboring cilia just generates local swirls. For example, $k_\varphi =3$ corresponds to a phase lag between neighboring cilia on the equator of $\Delta \Psi = k_\varphi 360^\circ /N_{\mathrm{eq}}$, which implies a large phase lag of $\Delta \Psi \simeq 150^\circ$ (for $N_{\mathrm{eq}}=7$). The rotational component of the propulsion also slightly reduces the component that contributes to the swimming velocity, compare Fig. 5a.

**Fig. 6:**
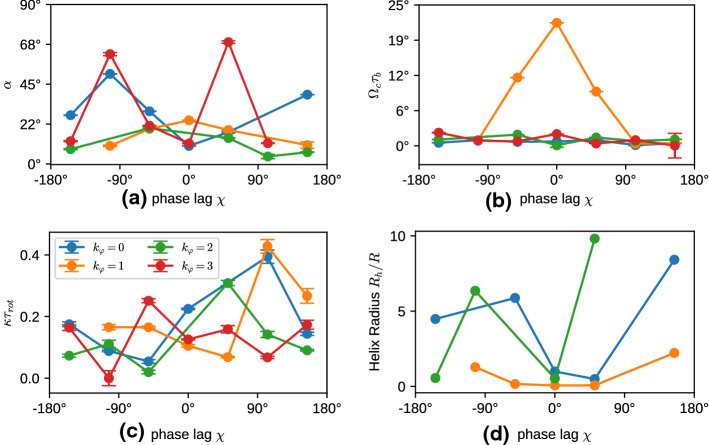
Swimming properties of a 5-7-5 swimmer with cilia beat in the longitudinal direction ($\theta _r=0$), for various phase lags $\chi$ and latitudinal modes $k_\varphi$. **a** Alignment angle $\alpha$; **b** Rotation frequency $\Omega _c$, normalized by the beat period $\tau$; **c** Inverse correlation time $\kappa$, normalized by the rotational diffusion time $\tau _{\mathrm{rot}} = 1/(2D_{\mathrm{rot}})$. **d** Helix radius $R_h$ in units of the body radius *R*, as obtained from Eq. (5)

For $k_\varphi \ge 1$, the chirality of the wave pattern not only induces a body rotation, but also implies a helical swimming trajectory [42, 52]. Consider a swimmer, for which the main body axis $\mathbf{n}$ rotates around a fixed axis $\mathbf{e}_\parallel$ in the laboratory reference frame with an opening angle $\alpha$. In the absence of translational and rotational noise, this corresponds to the trajectory of a perfect helix with axis $\mathbf{e}_\parallel$ and azimuthal direction $\mathbf{e}_{\perp }(t)$

3 $$\begin{aligned} \mathbf{n}(t)= & {} n_{\parallel } \mathbf{e}_\parallel + n_\perp \mathbf{e}_{\perp }(t) \nonumber \\ n_\parallel= & {} \cos \alpha \nonumber \\ n_\perp= & {} \sin \alpha \end{aligned}$$

where $\mathbf{e}_{\perp }(t)=(\cos (\Omega _c t), \sin (\Omega _c t))$ in Cartesian coordinates in the plane with normal vector $\mathbf{e}_\parallel$.

The opening angle $\alpha$, the rotation frequency $\Omega _c$, and swim velocity $v_n$, are related to the helix parameters—helix radius $R_h$, pitch length $P_h$, an helix angle $\alpha _h$—as

4 $$\begin{aligned} \cos (\alpha )^2= & {} P_h^2/[P_h^2 + 4\pi ^2 R_h^2], \nonumber \\ \alpha _h= & {} \arctan (2\pi R_h/P_h) \equiv \alpha , \end{aligned}$$

and

5 $$\begin{aligned} P_h = v_n \cos (\alpha )/\Omega _c, \ \ \ R_h = v_n \sin (\alpha )/(2\pi \Omega _c). \end{aligned}$$

For $\alpha =0$, the microswimmer moves on a straight lines, whereas for $\alpha =90^\circ$ it moves on a circle. In general, the directional auto-correlation function of a swimmer consists of two factors, the correlation due to the helical motion, which depends on the inclination angle $\alpha$, and an exponential decay due to thermal or active noise,

6 $$\begin{aligned} <\mathbf{n}(t) \cdot \mathbf{n}(t+\tau )> = \left( \cos ^2 \alpha + \sin ^2 \alpha \cos (\Omega _c \tau )\right) e^{-\kappa \tau }\nonumber \\ \end{aligned}$$

Correlation functions for fixed $\chi =-50^\circ$ and various $k_\varphi$ are shown in Fig. 5d. Results for the fitted parameters of the helical motion and the decay time $1/\kappa$ in Eq. (6) are displayed in Fig. 6. The calculation of these parameters requires very long simulation times of several hundred beat periods $\tau _b$ or more. Even in this case, trajectories are sometimes too short for a reliable parameter estimation, in particular for large decorrelation times $1/\kappa$.

**Fig. 7:**
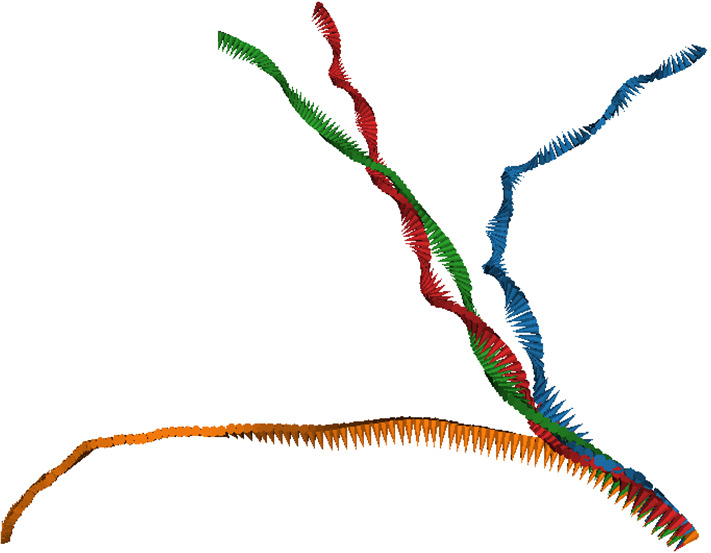
Helical trajectories for selected parameters of the metachronal beat, with phase lag $\chi =+105^\circ$. Trajectory colors indicate the latitudinal wave number, with $k_\varphi =0$ blue, $k_\varphi =1$ orange, $k_\varphi =2$ green, and $k_\varphi =3$ red. The small (body-fixed) arrows have their tip in the body center and their base on the equator to indicate rotation around the main body axis

For most cases, the helix angle $\alpha < 45^\circ$, which means that the constant term in Eq. (3) dominates over the oscillatory term, and thus the helix is thin and elongated. Only in a few cases, like $k_\varphi =3$, we find a nearly circular motion and a tightly wound helix. The circling frequency $\Omega _c$ is pronounced for $k_\varphi = 1$, and small phase lags $|\chi |$, which leads to a pronounced helical swimming trajectory, see Fig. 7. This is closely related to the large internal spinning frequency $\Omega _n$ (see Fig. 5c). For $k_\varphi \ge 2$, the circling frequency is typically very small, which is again related to the inefficiency of propulsion for short metachronal wave lengths. The data in Fig. 6 sometimes appear to have a larger “scatter” for different wave numbers $k_\varphi$ and different phase lags $\chi$. However, two points should be noticed. (i) We expect smooth curves for fixed $k_\varphi$ as a function of $\chi$ when $\chi$ is varied in small steps; however, we vary $\chi$ in rather large discrete steps of about $50^\circ$, which results in significantly different metachronal waves. (ii) Similarly, different $k_\varphi$ generate very different wave patterns, compare Figs. 3 and 4 for $k_\varphi =0$ and $k_\varphi =1$, respectively.

**Fig. 8:**
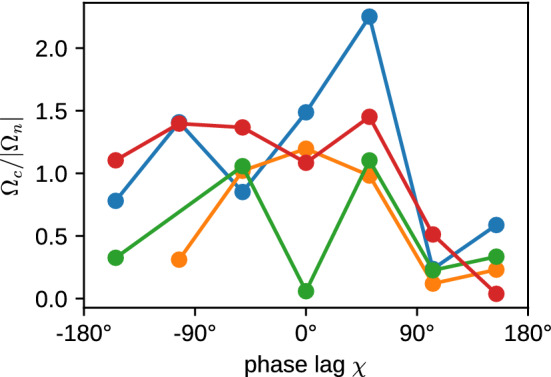
Ratio of circling frequency $\Omega _c$ and spinning frequency $\Omega _n$ for various latitudinal wave numbers $k_\varphi$. The color code is the same as in Fig. 6

The ratio $\Omega _c/|\Omega _n|$ is displayed in Fig. 8; it demonstrates that the circling and spinning frequencies are closely related. In many cases, $\Omega _c/|\Omega _n|\simeq 1$, which corresponds to a “twisted-ribbon-like” motion (or to a “tidal-locking-like” motion, as for the moon, which always presents the same side to the earth). However, there are also cases where $\Omega _c/|\Omega _n|$ is close to 0 or to around 2, which indicates that the two frequencies do not have to be locked always. An example for $\Omega _c/|\Omega _n|=0$ is a microswimmer which spins around its body axis, but swims on a straight trajectory.

It is interesting to note that the microswimmers with $k_\varphi =0$ do *not* have a vanishing circling frequency. The reason is that they are not perfectly axisymmetric and therefore have some inherent chirality, due to the non-symmetric location of the cilia on the body. For example, there is a particular longitude, where the cilia in the various rings are closest together, which generates a non-axisymmetric flow field. This leads to the helical trajectory displayed in Fig. 7, with a non-vanishing but very small $\Omega _c$. The spinning frequency $\Omega _n$ around the main body axis nearly vanishes in this case (see Fig. 5c), because for $\theta _r=0$ there is essentially no component of the cilia beat in the latitudinal direction.

Finally, the decay (or decorrelation) time $1/\kappa$ is determined by thermal fluctuations and Stokes friction, and possibly by active fluctuations generated by the beat. In the thermal case, we expect a rotational diffusion time $\tau _{\mathrm{rot}} = 1/(2D_{\mathrm{rot}})$ with rotational diffusion coefficient $D_{\mathrm{rot}}=k_BT/(8\pi \eta _f R^3)$, where $\eta _f$ is the fluid viscosity. The scaled decorrelation times $1/(\kappa \tau _{\mathrm{rot}})$ are mostly larger than 4, significantly larger than unity. This implies that (i) activity has a small effect on the rotational diffusion, and (ii) that the cilia contribute significantly to reduce the rotational diffusion by increasing the effective hydrodynamic radius. A factor 4 reduction is equivalent to a hydrodynamic radius $R_{hydro}= 1.6 R$, which is not implausible as the geometric radius from body center to cilia tips is 2.375*R*. The least persistent motion is observed for $k_\varphi =1$ and $\chi =100^\circ$, which indicates a rather strong active contribution to the noise, as might be expected for the strong wiggling motion for $k_\varphi =1$ (compare movie SM2).

We can now use the expressions (5) to extract the helix radius $R_h$ from the data presented in Figs. 5 and 6. The results are shown in Fig. 6d. They indicate that the helix radius $R_h$ is typically rather small, from essentially zero to just a few times the body radius *R*, with a few exceptions, like $k_\varphi =0$ and $\chi =150^\circ$. The helix pitch is usually a factor 10 larger than the radius, as typically $P_h/(2\pi R_h) = \cot (\alpha ) \gg 1$ (compare Eq. (4) and Fig. 6a). This is in good agreement with the trajectories displayed in Fig. 7.

Finally, we want to mention another important characteristics of a microswimmer, which is the *swimming efficiency*

7 $$\begin{aligned} \eta = \zeta _{st} \langle v_n \rangle ^2/\langle P \rangle \, \end{aligned}$$

where $\zeta _{st} =6\pi \eta _f R$ is the Stokes friction coefficient of the spherical body, and *P* is the dissipated power. Our simulations show that the dissipated power—obtained from the energy removed from the system through the thermostat—is nearly independent of the wave number $k_\varphi$ and the phase lag $\chi$. Thus, Eq. (7) implies that the functional dependence $k_\varphi$ and $\chi$ is dominated by the variation of the $\langle v \rangle$, compare Fig. 5a. The optimal efficiency of about 0.06% is therefore obtained for antiplectic waves in the range $-90^\circ< \chi < -40^\circ$ (slightly depending on the wave number $k_\varphi$). This value of the efficiency is somewhat smaller than for ciliated microswimmers with much shorter cilia [36], but of the same order of magnitude.

### The 5-7-5 swimmer with oblique power-stroke direction

**Fig. 9:**
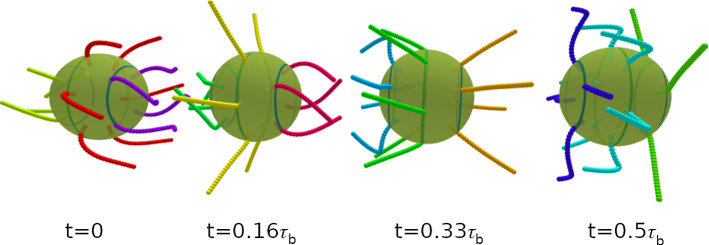
Temporal beat pattern of a 4-6-4 swimmer with cilia beat in an oblique direction ($\theta _r=22.5^\circ$), with latitudinal wave vector $k_\varphi =0$ and phase lag $\chi =-77^\circ$. The varying cilium color indicates the instantaneous stage in the beat cycle (compare Fig. 2). The progression of the beat is indicated by the time *t*, given in units of the beat period $\tau$. The motion of the swimmer is from left to right. The translational motion is not to scale. See also movie SM3 for an illustration of the swimming behavior

Except for the oblique propagation direction of the metachronal wave, there are other possibilities to achieve a chirality of the dynamic beat pattern of a ciliated microswimmer. One of these possibilities is to vary the power stroke direction away from the main body axis and rotate it in the local tangent plane to the left by a tilt angle $\theta _r$. For simplicity, we consider in this case only a metachronal wave in the main body direction, i.e., $k_\varphi =0$. An example for the beating dynamics with phase lag $\chi =-77^\circ$ and tilt angle $\theta _r=22.5^\circ$ is shown in Fig. 9.

**Fig. 10:**
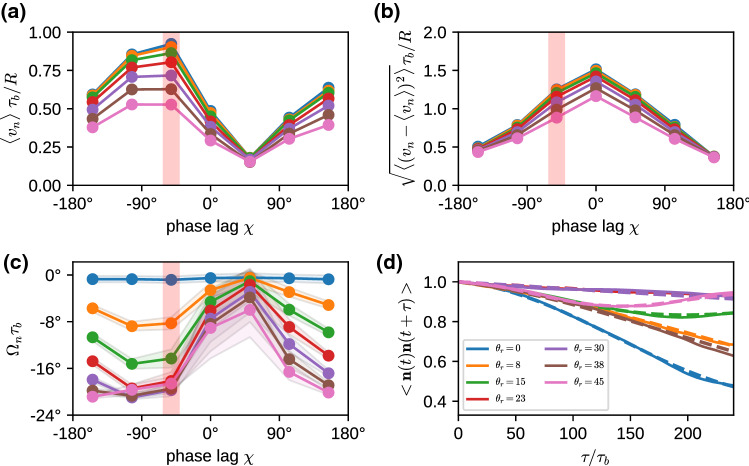
Swimming properties of a 5-7-5 swimmer with metachronal waves oriented along the main axis ($k_\varphi =0$) for various phase lags $\chi$ between successive rings and varying power stroke orientation $\theta _r$. **a** Average swimming velocity $<v_n>$. **b** Velocity fluctuations$\sqrt{<(v_n - <v_n>)^2>}$ around the average. **c** Rotational velocity $\Omega _n$ around the main body axis. **d** Auto-correlation function $<\mathbf{n}(t) \cdot \mathbf{n}(t+\tau )>_t$ for swimmers with phase lag $\chi =-50^\circ$ [indicated by red stripe in **(a**–**c)**]and varying $k_\varphi$. The dashed lines are fits to the auto-correlation function Eq. (6) (see text)

Results for the swimming properties with oblique power-stroke direction are displayed in Fig. 10. The results for the swimming velocity, velocity fluctuations, and rotational motion are qualitatively similar as for the oblique metachronal wave. There is again a “sinusoidal” dependence of the velocity $\langle v_n \rangle$ on the phase lag $\chi$, with a maximum for at $\chi =-50^\circ$, i.e., for an antiplectic metachronal coordination, see Fig. 10a. However, there are also several pronounced qualitative and quantitative differences. The velocity decreases with increasing $\theta _r$, because an increasing fraction of the beat is employed for body rotation rather than forward propulsion. Velocity fluctuations $\sqrt{\langle (v_n - \langle v_n \rangle )^2 \rangle }$ peak for synchronously beating cilia for all $\theta _r$, as all cilia beat in synchrony in this case (with $k_\varphi =0$), see Fig. 10b. The body rotation is much more pronounced for all $\theta _r>0$, see Fig. 10c, compared to the case of oblique metachronal wave with $\theta _r=0$. Interestingly, the body rotation becomes very slow for $\chi \simeq 50^\circ$ for all $\theta _r>0$. This minimum of spinning frequency $\Omega _n$ correlates with the minimum of propulsion velocity $v_n$, i.e., weak propulsion is accompanied by slow spinning. Correlation functions for fixed $\chi =-50^\circ$ and various $\theta _r$ are shown in Fig. 10d.

**Fig. 11:**
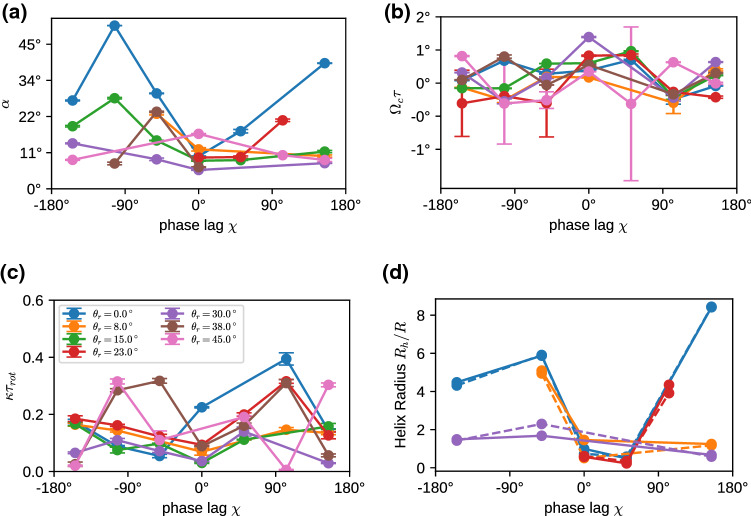
Swimming properties of a 5-7-5 swimmer with cilia beat in the longitudinal direction ($\theta _r=0$), for various phase lags $\chi$ and latitudinal modes $k_\varphi$. **a** Alignment angle $\alpha$; **b** Rotation frequency $\Omega _c$, normalized by the beat period $\tau$; **c** Inverse correlation time $\kappa$, normalized by the rotational diffusion time $\tau _{\mathrm{rot}} = 1/(2D_{\mathrm{rot}})$, with $D_r=k_BT/(8\pi \eta _f R^3)$, where $\eta _f$ is the fluid viscosity. **d** Helix radius $R_h$ in units of the body radius *R*, as obtained from Eq. (5)

Results for the fitted parameters of the helical motion and the decay time $1/\kappa$ in Eq. (6) are displayed in Fig. 11. For most cases, the helix angles $\alpha$ are small, around $10^\circ$ to $25^\circ$, which implies that the constant term in Eq. (3) dominates over the oscillatory term, and the helices are very thin and elongated. The circling frequencies $\Omega _c$, displayed in Fig. 11b, are generally very small, which implies that cilia beat orientation with $\theta _r>0$ results more in spinning than in circling. Scaled decorrelation times $1/\kappa \tau _{\mathrm{rot}}$ are typically larger than 4, which indicates a large hydrodynamic radius, in agreement with the conclusions of Sect. 3.1.

We can use again the expression (5) to extract the helix radius $R_h$ from the data presented in Figs. 10 and 11. The results are shown in Fig. 11d. They indicate that similarly as for the case $\theta _r=0$ displayed in Fig. 6d, the helix radius $R_h$ is typically rather small, from nearly zero to just a few times the body radius *R*. The helix pitch is typically about a factor 10 larger than the radius.

### Variation of cilia number

**Fig. 12:**
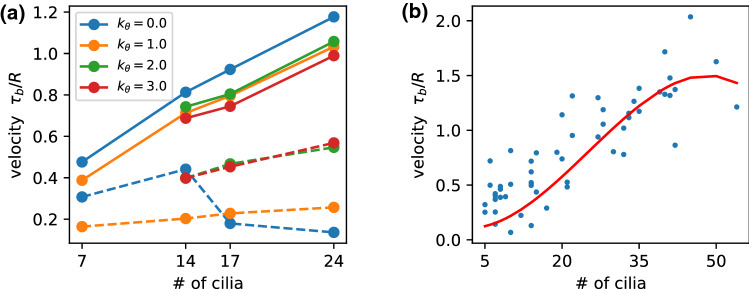
Swimming velocity for microswimmers with various numbers of cilia. **a** With a predefined metachronal wave with various latitudinal wave numbers $k_\varphi$, as indicated. Full (dashed) lines show that maximum (minimum) velocity obtained by variation of the phase lag $\chi$. **b** With self-organized metachronal waves and random spatial arrangement. The red line is a spline fit to different cilia arrangements. See also movie SM4 for an illustration of the beat and swimming behavior

We have considered so far a spherical microswimmer with a fixed number $N=5+7+5=17$ cilia. It is now of course interesting to see how the swimming behavior, in particular the swimming velocity, depends on the number of cilia. For the swimmer with imposed metachronal wave and $\theta _r=0$, as described in Sect. 3.1, the results are shown in Fig. 12a, both for the maximum and minimum velocity obtained for various phase lags $\chi$. In general, the maximum velocity is found to increase with cilia number, consistent with the results of Ref. [35] for high cilia numbers. For all cilia numbers considered, the highest velocity is obtained for $k_\varphi =0$, i.e., for a wave direction along the main body axis, in agreement with our arguments in Sect. 3.1 above. In contrast, the variation of the minimum velocity on cilia number is much less pronounced.

We want to compare this result with the case of a *self-organized metachronal wave*—similar to what is studied in Ref. [17] for planar cilia arrays. The cilia are distributed randomly, with a minimum-separation requirement to neighboring cilia, and as homogeneously as possible on the body surface. We consider several different realizations, so that the variance of the results for the same cilia number can be taken as a measure for the sensitivity of the swimming velocity on the spatial distribution. It is important to emphasize that a perfect equidistant distribution of anchoring point on a spherical cannot be achieved, so that some spatial inhomogeneity is unavoidable [53]. Furthermore, the “hairy-ball” theorem implies that the field of cilia-beat directions must have at least two singularities—which we usually place at the poles. For a random distribution, however, one of the cilia may end up by chance very close to the pole, which then has a very strong effect of the swimming direction. We avoid this case by manual selection.

Results are shown in Fig. 12b. Again, the velocity increases with cilia number, but seems to level off at about 40 cilia. This saturation is not too surprising, as with increasing cilia density, the effect of each individual cilium on the propulsion diminishes due to the interaction with the neighbors [17, 36]. For *N* cilia on the surface, the average distance *d* between them is approximately determined by $d \simeq (4\pi R^2/N)^{1/2}$, which yields $d/L \simeq 1/3$ for $N=40$ and cilia length $L=1.375\,R$. This estimate is also comparable to the optimal cilia distance of d/L=0.23 found in Ref. [36].

The results for the self-organized metachronal wave on a spherical body can now be compared with those on a planar substrate as a function of *d*/*L*. For the planar case, the fluid transport velocity was found to scale as $v_{fluid} \sim (d/L)^{-\gamma }$ with $\gamma \simeq 1.4$ [17]. This implies a dependence of the swimming velocity $v_{swim}$ on cilia number as $v_{swim} \sim N^{\gamma /2}$, i.e., a behavior somewhere between linear and square root, which seems not inconsistent with the numerical results of Fig. 12b. The simulation results of Ref. [35] for microswimmers with large cilia numbers (in the range $N=20$ to $N=320$) also show a sublinear dependence of $v_{swim}$ on *N*, which a nearly linear dependence for intermediate values of the phase lag $\chi$, and a strongly sublinear dependence for $\chi =0$.

It is also interesting to note that the results for cilia arrays on a planar substrate indicate that $d/L=1/6$ is in the regime where transport with metachronal coordination is much more efficient than synchronous beating [17]—in qualitative agreement with the results displayed in Fig. 12a.

## Summary and conclusion

The dynamics and motion of multi-ciliated microswimmers with a spherical body and a small number *N*, in the range $5< N <50$, of long cilia, with length *L* about a factor 1.4 larger than the body radius, has been investigated by mesoscale hydrodynamics simulations. A metachronal wave is imposed for the cilia beat, for which the wave vector has both a longitudinal, $k_\theta$, and a latitudinal, $k_\varphi$ component. The dynamics and motion is characterized by the swimming velocity $v_n$ along the main body axis, the variance of the velocity averaged over a full beat cycle, the spinning velocity $\Omega _n$ around the main body axis, as well as the parameters of the helical trajectory, which are the circling velocity $\Omega _c$, the helix angle $\alpha$, the helix radius $R_h$ and pitch $P_h$.

Our simulation results show that the microswimmer motion strongly depends on the latitudinal wave number $k_\varphi$ and the longitudinal phase lag $\chi =k_\theta \cdot \, 45^\circ$. We find, not unexpectedly, that the microswimmers usually spin around their own axis, and swim on helical trajectories. It is important to notice that spinning and helical motion are not necessarily correlated, as a spinning particle can move on a perfectly straight trajectory. However, chirality in the metachronal beat pattern generically generates helical trajectories. In most cases, the helices are found to be thin and stretched, i.e., the helix radius $R_h$ is about an order of magnitude smaller than the pitch. An interesting result is also that the rotational diffusion of the microswimmer is significantly smaller than the passive rotational diffusion of the body alone. This indicates that active contributions to rotational diffusion are small, and that the extended cilia make a pronounced contribution to the hydrodynamic radius.

The swimming velocity $v_{swim}$ increases with the number *N* of cilia on the body. Our simulation results indicate a slightly sublinear dependence on *N*. The comparison with the transport velocity of planar cilia arrays on the cilia separation predicts a dependence $v_{swim} \sim N^{0.7}$. Our simulation results for self-organized metachronal waves are not inconsistent with such a relation.

Finally, it is interesting to note that already a relatively small number of about ten cilia, beating with a phase lag in the form of a metachronal wave, are sufficient to generate a steady propulsion and smooth swimming motion—in contrast to the strongly oscillatory motion of Chlamydomonas [54, 55] with its two cilia beating in synchrony. Such a smooth swimming motion can be of significant benefit for marine microorganisms, because large disturbances can be exploited by predators to locate their prey [56].

### Supplementary Information

Below is the link to the electronic supplementary material.

Supplementary material 1 (pdf 43 KB)

Supplementary material 2 (mp4 3271 KB)

Supplementary material 3 (mp4 2488 KB)

Supplementary material 4 (mp4 3295 KB)

Supplementary material 5 (mp4 5721 KB)

## Figures and Tables

**Fig. 13:**
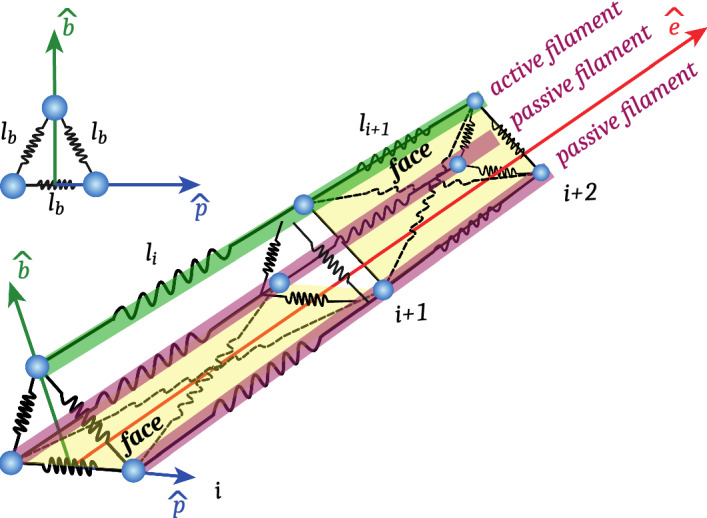
Sketch of the cilium model in the local reference frame ${\hat{e}},{\hat{b}},{\hat{p}}$- The cilium consists of one active rod green and two passive ones red, forming an equilateral triangle. It’s main axis is oriented along ${\hat{e}}$ and it beats in the ${\hat{b}}$ direction. Each bead blue is connected by several springs black to its neighboring beads. Two diagonal springs connect the edges of each face. Not that they are only shown for the two yellow-marked faces to improve readability. Segments along ${\hat{e}}$ are labeled in ascending order, starting at $i=0$ and have a distance of $l_b=0.5~a$

**Table 1:**
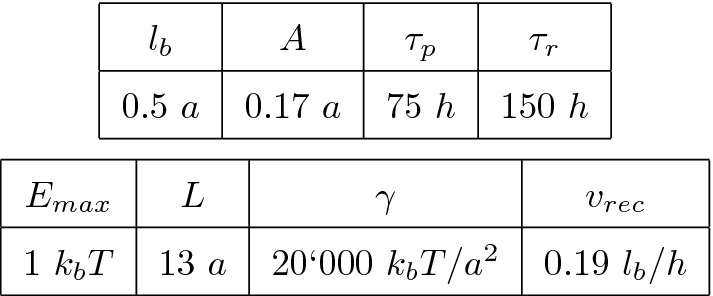
Model parameters (given in MPC units of thermal energy $k_BT$, collision cell size *a*, and collision time *h*) used to generate the beat pattern of the cilium in the simulations

**Fig. 14:**
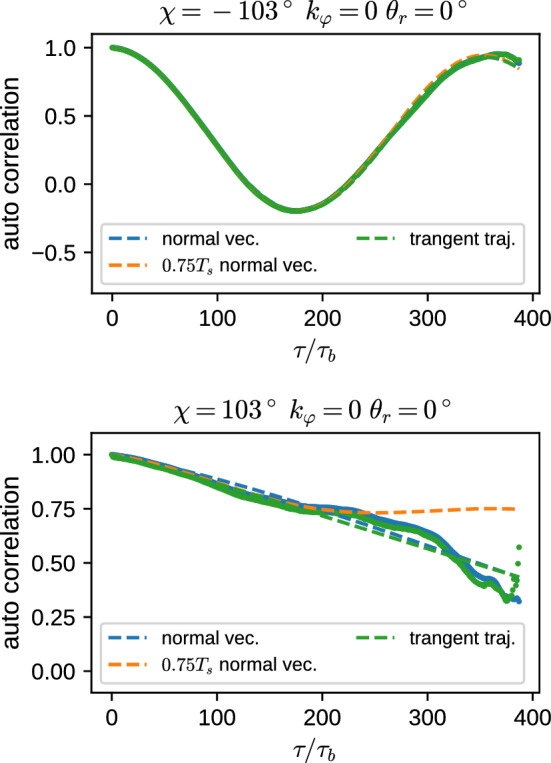
Fit results of the temporal auto-correlation function which characterizes the helical trajectory. Either the auto-correlation function of the unit normal vector $\mathbf{n}$ of the main body axis is employed, or of the tangent vector along the smoothed center-of-mass trajectory, both with a temporal delay $\tau$. Simulation data are shown as solid lines, fits to Eq. (6) as dashed lines

## Data Availability

Data, simulation code, and some data-analysis tools are available on zenodo, see https://doi.org/10.5281/zenodo.4733431

## Appendix A. Ciliary beat

The cilium is considered as a semi-flexible filament, which is modeled by point particles (beads) that are interconnect by springs. Beads are arranged in three linear chains that form a bundle with cross section of an equilateral triangle (see Fig. 13). Each chain consists of $N_c=26$ individual beads equally spaced at a distance of 0.5 *a*, where *a* is the linear MPC collision-cell size. Springs with equilibrium length $l_b$ connect each bead to its neighboring beads along the line. Two diagonal connection on each face provide lateral stability (see Fig. 13). The forces along the active chain are generated by changing the equilibrium length of the springs along the chain [17].

The beat pattern is generated by a heuristic model for the time evolution of bond forces along the chain. In this description, the beat can be controlled by a few key parameters and allows adaptation to external flows. The beat is regulated by making the equilibrium curvature along the cilium depend on a pivot point $i_0$. Thus, the bond length along the active filament varies as

A.1 $$\begin{aligned} l_i(t)= & {} l_b + \delta l_i(t) \nonumber \\ \delta l_i(t)= & {} 0.5 A \left( 1 - \frac{i - n_0 - 1}{N_F -n_0 - 1} \right) ^{2.5} \nonumber \\&\times \left( 1 - \frac{1}{i_0-i-1} \right) \ \ \ \ \text {for } i < i_0 -2 \nonumber \\ \delta l_i(t)= & {} - \frac{2A}{(i - i_0)^2+1}\ \ \ \ \ \text {for } i \ge i_0 - 2, \end{aligned}$$

where *i* is the segment number which varies from $n_0$ to $L/l_b$. The first three beads of each linear chain are passive and stay at the bond-length value $l_{i<n_0} = l_b$. Therefore, we set $n_0=3$.

The difference between power and recovery stroke depends on the pivot-point position $i_0$. During the power stroke, the pivot point is set to the first point of active beating $n_0$. It stays at this position, until the power stroke time $\tau _p$ has passed. Then, during the recovery stroke, the pivot-point $i_0$ moves along the cilium with a constant velocity $v_{\mathrm{rec}}$ until the recovery-stroke time $\tau _r$ has passed. Together, this results in a beat as displayed in Fig. 2, with a constant beat period $\tau _b = \tau _p + \tau _r$.

In addition to the global beat dynamic, the dynamics of the beat pattern is limited by the maximum energy $E_{\mathrm{max}}$ each motor can inject into the system. If the bond-length change $\delta l_i$ compared to the current distance between the beads exceeds the maximal energy $E_{\mathrm{max}}$ a motor can exert, the motor stall and $\delta l_i$ is reduced such that a maximum energy of $E_{\mathrm{max}}$ is not exceeded. Due to the thermal noise in the system, the actual distance between two beads fluctuates as well. Although we do allow for “helpful” fluctuations, we prohibit the “obstructive” fluctuations by clipping the bond length at the previous value $\delta l_i(t+1) = \delta l(t)$. This update scheme ensures step-wise directional movement similar to an active Brownian ratchet.

The parameters of the cilia beat pattern are specified in Table 1.

## Appendix B Parameter extraction from correlation functions

Due to the long time scales and the inherent noise of the auto-correlation function, it is difficult to extract parameters reliably. The signal-to-noise ratio is particularly high in the limit where the time scales $1/\Omega _c$ and $1/\kappa$ approach the total simulation time $T_s$.

Due to the approximate rotational symmetry of the microswimmer, we assume that the tangent vector to its center-of-mass trajectory is along the main body axis $\mathbf{n}$. This allows us to access the correlation functions, which determine the trajectory in two different ways. Two examples for the quality of the fit results are shown in Fig. 14. In order to estimate the quality of the fits, we employ the difference between two different—although related—auto-correlation functions which characterize the helical swimming motion. On the one hand, we use the correlation function $\langle \mathbf{n}(t) \cdot \mathbf{n}(t+\tau ) \rangle _t$ of the main body axis, as discussed in the main text; on the other hand, the correlate the tangent vectors of the smoothed center-of-mass trajectory with a window of $\tau$.

For a fixed total simulation time $T_s$, the error of the auto-correlation function increases proportional to $\sqrt{\tau /T_s}$, which is used as a weight factor for the fit. As a second error estimate, we employ the difference between a fit of the full trajectory with $\tau \le T_s$, and a fit for $\tau < 0.75~T_s$. Parameters, where the fit does not converge, or where the difference between the parameter obtained from the full fit and a partial fit exceeds the error threshold displayed in Table 2, are omitted and are not shown in the plots.

Furthermore, the microswimmer can perform motions which more complex than a simple helix, e.g., a nutation-like motion. This is not captured by our analytic formula, Eq. (6). We thus omit all results from fits with a too large mean squared deviation.

**Table 2:** Error thresholds for the fits of the auto-correlation functions

Parameter	Threshold
$\kappa$	$0.2/\tau _{\mathrm{rot}}$
$\Omega _c$	$\pm 5^\circ /\tau _b$
$\alpha$	$\pm 25^\circ$
