# Supplementary material for: Multi-ciliated microswimmers–metachronal coordination and helical swimming
Source: Eur Phys J E Soft Matter. 2021 Jun 8;44(6):76. doi: 10.1140/epje/s10189-021-00078-x (PMC8187229; doi:10.1140/epje/s10189-021-00078-x)
Supplement: Supplementary file 1 — Supplementary material 1 (pdf 43 KB) [file 10189_2021_78_MOESM1_ESM.pdf]

Multi-Ciliated Microswimmer – Metachronal Coordination and Helical Swimming

Sebastian Rode, Jens Elgeti, and Gerhard Gompper

Movie Captions:

Supplementary Movie SM1:

Visualization of the swimming motion and cilia beat pattern of a 4-6-4 swimmer with imposed metachronal wave (χ=-77°, θ_r=0) with latitudinal wave vector k_ϕ=0. The time dependence of the cilium color indicates the instantaneous beat phase (compare Fig. 2 of the main text). Note the antiplectic wave traveling from the back to the front and the nearly straight forward motion.

Supplementary Movie SM2:

Swimming behavior and beat pattern of a 4-6-4 swimmer with imposed beat pattern (χ=-77°, θ_r=0) with latitudinal wave vector k_ϕ=1. The time dependence of the cilium color indicates the instantaneous beat phase (compare Fig. 2 of the main text). Note the antiplectic wave (with a small laeoplectic component) traveling from with an angle relative to the power-stroke direction. This causes precession and nutation-like motion of the microswimmer, which eventually leads to a helical trajectory.

Supplementary Movie SM3:

Visualization of the swimming behavior and beat pattern of a 4-6-4 swimmer with imposed beat pattern (χ=-77°, θ_r=22°) with latitudinal wave vector k_ϕ=0. The time dependence of the cilium color indicates the instantaneous beat phase (compare Fig. 2 of the main text). Note the antiplectic wave (with a small laeoplectic component) traveling from the back to the front. In this case, the cilia are rotated, such that the power stroke points no longer along the main body axis, but has a lateral component. This causes a rotation of the microswimmer, in particular during the power stroke of the equatorial cilia.

Supplementary Movie SM4:

Swimming behavior and beat pattern of a spherical microswimmer with 24 cilia, arranged in three rings of 8 cilia each. Each point particle constituting the cilia or the swimmer body is rendered as a small sphere for better visualization. The active filament of each cilium is marked blue. The cilium beat is not prescribed in this case, but can adapt to local flow forces. All cilia start in synchrony, but develop a metachronal wave by self-organization after a few beats.
